# Integrated management of cryptococcal meningitis and concurrent opportunistic infections to improve outcomes in advanced HIV disease: a randomised strategy trial

**DOI:** 10.12688/wellcomeopenres.19324.1

**Published:** 2024-01-08

**Authors:** Jayne Ellis, Laura Nsangi, Ananta Bangdiwala, Gila Hale, Jane Gakuru, Enock Kagimu, Timothy Mugabi, Enos Kigozi, Asmus Tukundane, Michael Okirwoth, Tadeo Kiiza Kandole, Fiona Cresswel, Thomas S. Harrison, David Moore, Katherine Fielding, David Meya, David Boulware, Joseph N. Jarvis

**Affiliations:** 1 College of Health Sciences, Makerere University, Infectious Diseases Institute, Kampala, Uganda; 2 Clinical Research Department, London School of Hygiene and Tropical Medicine, London, UK; 3 University of Minnesota Twin Cities, Minneapolis, Minnesota, USA; 4 MRC Centre for Medical Mycology, University of Exeter, Exeter, England, UK; 5 Centre for Global Health, Institute for Infection and Immunity, St George's University of London, London, UK; 6 Botswana Harvard AIDS Institute Partnership, Gaborone, Botswana

**Keywords:** HIV, cryptococcus, meningitis, TB, preventive therapy

## Abstract

**Background:** Mortality associated with HIV-associated cryptococcal meningitis remains high even in the context of clinical trials (24–45% at 10 weeks); mortality at 12-months is up to 78% in resource limited settings. Co-prevalent tuberculosis (TB) is common and preventable, and likely contributes to poor patient outcomes. Innovative strategies to increase TB preventative therapy (TPT) provision and uptake within this high-risk group are needed.

**Protocol:** The IMPROVE trial is a nested open label, two arm, randomised controlled strategy trial to evaluate the safety (adverse events) and feasibility (adherence and tolerability) of two ultra-short course TPT strategies, in the context of recent diagnosis and treatment for cryptococcal meningitis. We will enrol 205 adults with HIV-associated cryptococcal meningitis from three hospitals in Uganda. Participants will be randomised to either inpatient initiation (early, week 2) or outpatient initiation (standard, week 6) of 1HP (one month of isoniazid and rifapentine). Participant follow-up is to include TB screening, pill counts and tolerability reviews on alternate weeks until week-18. The trial primary endpoint is TB-disease free 1HP treatment completion at 18-weeks, secondary endpoints: 1HP treatment completion, 1HP discontinuation, grade ≥3 adverse events and serious adverse events, drug-induced liver injury, incident active TB, 18-week survival; rifapentine, fluconazole and dolutegravir concentrations will be measured in a drug-drug interaction sub-study of 15 eligible participants.

**Discussion:** The IMPROVE trial will provide preliminary safety and feasibility data to inform 1HP TPT strategies for adults with advanced HIV disease and cryptococcal meningitis. The potential impact of demonstrating that inpatient initiation of 1HP TPT is safe and feasible amongst this high-risk subpopulation with advanced HIV disease, would be to expand the range of clinical encounters in which clinicians can feasibly provide 1HP, and therefore increase the reach of TPT as a preventative intervention.

**ISRCTN registration:** ISRCTN18437550 (05/11/2021)

## Introduction

Cryptococcus is the most common cause of HIV-associated meningitis globally, accounting nearly 20% of all AIDS-related deaths ^1^. Despite antifungal therapy, 10-week mortality in sub-Saharan Africa remains between 24 and 45%, even in the context of clinical trials ^2– 4^. Due to advanced immunosuppression, mortality continues beyond hospital discharge particularly in those with CD4<50 cells/µL ^5^, and mortality at 12-months after cryptococcal meningitis diagnosis is between 40% and 78% in resource limited settings ^6^. The key drivers of this persistently high mortality are not known; however, recent data suggest that co-prevalent opportunistic infections including tuberculosis are common and likely contribute to poor patient outcomes ^7– 9^. A broader pre-emptive anti-infective package now warrants investigation.

Tuberculosis (TB) is treatable and preventable, yet it remains the most frequent cause of AIDS-related deaths worldwide ^10, 11^. All people living with HIV (PLHIV) should be systematically screened for active TB disease at every clinical encounter; and following exclusion of active TB disease, TB preventive therapy (TPT) should be provided for all PLHIV, irrespective of anti-retroviral therapy (ART) status and CD4 count ^11^. Despite clear recommendations from the World Health Organization (WHO), and robust data that TPT prevents TB disease and deaths ^12– 14^, provision of TPT has been sub-optimal globally. In 2019, of the 38 high TB and TB/HIV burden countries only 23 reported provision of TPT for those receiving ART; coverage varied considerably from less than 1% in Thailand to 89% in Zimbabwe ^10^. Dramatic scale up of TPT for PLHIV is one of the WHO’s key pillars to meet the 2030 and 2035 End TB Strategy targets ^15^. Innovative delivery strategies to increase TPT provision are urgently needed.

Barriers to TPT implementation are multi-factorial and include concerns about TPT adherence, loss to follow-up, and drug toxicity ^13^. These concerns are pertinent given the historically long duration of TPT regimens (six or nine months of isoniazid (6H/9H). In 2019 however, the landmark BRIEF TB/A5279 trial demonstrated that one month of rifapentine plus isoniazid (1HP) was non-inferior to 9H for preventing active TB disease in PLHIV. Additionally, treatment completion rates were the highest ever reported in a TPT trial (97%), with a lower incidence of adverse events in the 1HP arm ^13^. 1HP is a short, efficacious, well-tolerated, and safe TPT regimen, and was endorsed by WHO as a TPT option in 2020 ^11^. 1HP – if combined with innovative delivery strategies to increase TPT uptake – offers a major potential breakthrough in the prevention of TB amongst PLHIV globally.

Ultra-short course 1HP TPT may have particular advantages over longer course TPT for patients with advanced HIV disease (AHD), in whom risk of TB disease is greatest. In the context of AHD, expedited completion of TPT has clear benefits with respect to pill burden, drug-drug interactions (DDIs) and in rapid sterilisation of latent TB infection (LTBI) ^11, 16^. TPT with 1HP is of particular interest in cryptococcosis. In HIV-associated cryptococcal meningitis, ART initiation is delayed due to the risk of cryptococcal-immune reconstitution inflammatory syndrome (IRIS) ^3^. The risk of unmasking TB-IRIS, however, remains following ART initiation at 4–6 weeks, with most incident IRIS events occurring within the first month of ART initiation ^12, 17, 18^. Completion of 1HP TPT prior to ART initiation has the potential to reduce incidence of TB-IRIS, active TB disease, and TB deaths amongst this subpopulation ^12, 19^.

The IMPROVE trial will evaluate the safety and feasibility of two strategies for the delivery of 1HP TPT in adults with AHD and cryptococcal meningitis: inpatient initiation (early, during week 2 of anti-fungal therapy for cryptococcosis) or outpatient initiation (standard, during week 6 of anti-fungal therapy for cryptococcosis) of 1HP. Currently, the majority of TPT globally is provided in the outpatient setting. We propose that initiation of 1HP prior to hospital discharge has the potential to increase the reach of TPT as an intervention, and to reduce losses from the LTBI preventive care cascade (identification of at-risk populations, exclusion of active TB, provision of TPT, monitoring for adverse events, adherence and completion of treatment) ^20^. The potential benefit of inpatient initiation of TPT, however, should be carefully balanced against the risk for drug-related adverse events including risk of DDIs with antifungals or ART, and poor adherence due to additional pill burden. The optimal TPT strategy to prevent TB disease in HIV-associated cryptococcal meningitis therefore needs to be determined.

## Study design

The IMPROVE trial is an open label, two arm, randomised controlled strategy trial to evaluate the safety and feasibility of two 1HP TPT strategies for adults with HIV-associated cryptococcal meningitis. The IMPROVE trial is nested within an observational cohort study screening for concurrent opportunistic infections (OIs) in patients undergoing treatment for cryptococcal meningitis.

**Figure 1.**
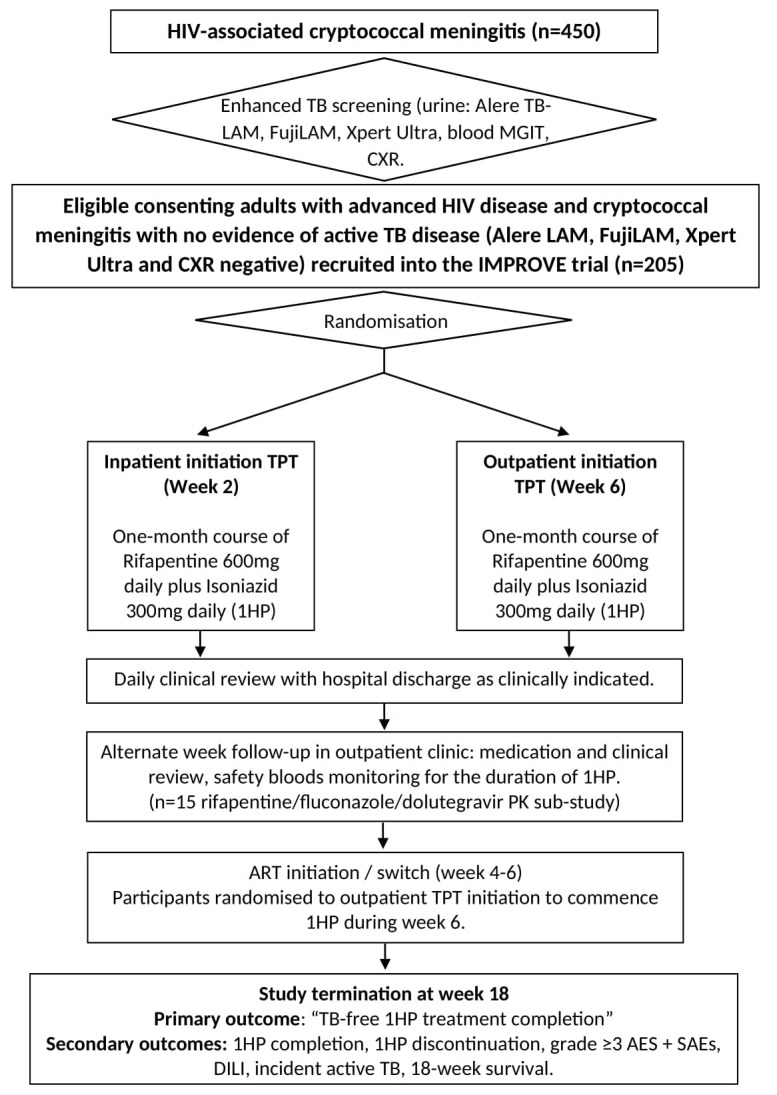
IMPROVE study flow chart. MGIT=mycobacteria growth inhibitor tube; CXR = Chest X-ray; TPT=TB preventative therapy; AEs=adverse events; SAEs=serious adverse events.

All consenting adults (≥18 years) will be screened for active TB disease with urine (Alere TB-LAM, Fuji SILVAMP TB LAM and Xpert Ultra), blood (mycobacteria growth inhibitor tube (MGIT)) culture and chest x-ray (CXR) as part of the ongoing prospective observational cohort study. Study participants in whom active TB disease has been systematically excluded will be randomized (1:1) to inpatient initiation or outpatient initiation 1HP TPT ( Figure 1).

This trial has been registered on ISRCTN (ISRCTN18437550) on 5 ^th^ November 2021. This article follows the SPIRIT guidelines ^21^.

## Hypothesis

Our primary hypothesis is that inpatient initiation of 1HP TPT will be non-inferior to outpatient initiation of 1HP TPT with respect to “TB-disease free 1HP treatment completion”, and that inpatient 1HP TPT is safe (adverse events) and feasible (adherence and tolerability) in patients with HIV-associated cryptococcal meningitis.

### Primary objective

- To generate evidence on the safety (adverse events) and feasibility (adherence and tolerability) of 1HP TPT amongst adults with HIV-associated cryptococcal meningitis.

### Secondary objective

- To generate preliminary data on potential secondary benefits (reduced loss to follow-up, reduced active TB disease, reduced mortality) of inpatient initiation of 1HP TPT as compared to outpatient initiation of 1HP TPT amongst adults with HIV-associated cryptococcal meningitis.

## Study setting

The trial will be set in three hospitals in Uganda: Kiruddu National Referral Hospital, Mulago National Referral Hospital, Kampala and Mbarara Regional Referral Hospital. The study population will be HIV-positive adults (≥18 years), diagnosed with HIV-associated cryptococcal meningitis.

### Primary endpoint

TB-disease free 1HP treatment completion at 18-weeks (after cryptococcal meningitis diagnosis and commencement of anti-fungal therapy). Treatment completion is defined as participant reported adherence to >90% of the study medications, to be completed within 6-weeks from treatment initiation. TB-disease free at 18-weeks is defined as not receiving a diagnosis of active TB disease for the duration of the trial during the 18-week study period.

### Secondary endpoints

**1.**  1HP treatment completion at 18-weeks.
**2.**  1HP discontinuation of the study drugs for ≥ 5 consecutive days for any reason.
**3.**  Grade ≥3 adverse events (AEs) and serious adverse events (SAEs).
**4.**  Drug-induced liver injury defined as elevation of blood transaminase (ALT) alone ≥ 5x ULN (or ALT ≥ 3x ULN if bilirubin abnormal) or alkaline phosphatase (ALP) alone ≥2x ULN.
**5.**  Incident active TB.
**6.**  18-week survival.
**7.**  Fluconazole, rifapentine and dolutegravir pharmacokinetics (PK)/ pharmacodynamics (PD) analyses (N=15).

### Inclusion criteria

Consecutive hospitalised adults (≥ 18 years) diagnosed with HIV-associated cryptococcal meningitis (confirmed by CSF CrAg testing) will be included in the study. We will include both initial and relapse cryptococcal meningitis episodes. Participants must be HIV-positive. Participants must provide written informed consent or, if unable to consent, have a next of kin who agrees to the patient participating in the study, providing written consent. 

### Exclusion criteria

Any patient with active TB disease (as evidenced by any positive TB screening test or taking TB therapy at time of screening) will not be eligible for enrolment. In addition, patients with clinical jaundice, abnormal liver function tests (bilirubin > 3.5 mg/dL or alanine aminotransferase (ALT) >200 IU/L), known chronic liver disease, active hepatitis B infection (defined as hepatitis B surface antigen positive) or presenting with a clinical syndrome which in the opinion of the attending clinician, puts the patient at significant risk if he/she were to participate in the 1HP trial will be excluded from the trial. Patients taking any contra-indicated medications including protease inhibitors will not be eligible for inclusion. Hypersensitivity to rifamycins or isoniazid is an exclusion criterion, as are pregnancy and breast feeding.

## Consent

Given the nature of cryptococcal meningitis it is anticipated that some patients will lack capacity to consent for themselves. Written informed consent to enter the trial will therefore be obtained from participants or, in the case of those lacking capacity to consent, from next of kin with legal responsibility. Illiterate volunteers will be asked to have a witness present (friend, family or another member of staff independent of the study team) to witness the discussion and thumbprint consent.

The aims, implications, potential benefits and risks associated with the study will be explained in full to all potential participants and/or the next of kin. It will be made clear to potential participants that refusal to participate in the study will not jeopardize their clinical care, and it will be made clear that consent is entirely voluntary and can be withdrawn at any time. Participants enrolled via surrogate consent will be re-consented as soon as their mental status improves and they regain the capacity to consent, with care taken to ensure they understand that they are free to withdraw from the study and if they do so this will not jeopardise their future care.

Original signed consent forms will be kept by the investigator, participants will be given a copy of the signed/thumb-printed consent form and a participant information sheet. The patient information sheet and consent form can be found as *Extended data* ^21^.

### Withdrawals

Participants may withdraw from the study at any time by withdrawing their consent. Assessment of vital status up until 18-weeks (secondary outcome) will continue via telephone calls at a minimum, unless consent is completely withdrawn.

### Randomisation and treatment allocation

Following screening and enrolment, participants will be randomised individually, based on random block sizes, using a computer-generated programme to either inpatient initiation (early, week 2) or outpatient initiation (standard, week 6) 1HP TPT. Participants will be randomized on the planned day of discharge from hospital; the specific timing of randomisation will be participant specific as it will depend on the clinical condition of the patient as monitored by the attending study physician and their time of hospital discharge. In instances, where the participant remains an inpatient for ≥14 days, randomisation will occur on day 14 rather than on the planned day of discharge. The trial pharmacist at each study site is responsible for conducting the randomisation by sequentially drawing sealed envelopes that contain the treatment assignment for each enrolled patient.

### Interventions

A summary of study interventions including timings are detailed below in the schedule of events table ( Table 1).

**Table 1.** Schedule of events table.

	Day 1	Wk 1	Wk 2	Wk 3	Wk 4	Wk 5	Wk 6	Wk 7	Wk 8	Wk 9	Wk 10	Wk11	Wk 12	Wk 13	Wk 14	Wk 15	Wk 16	Wk 17	Wk 18
Cryptococcal meningitis diagnosed	X																		
Commence anti-fungals ^1^	X																		
Inpatient TB screening package ^2^	X	X	X																
IMPROVE 1HP RCT enrolment		X	X																
Randomisation ^3^			X																
1HP initiation (early inpatient arm) ^4^			X																
Hospital discharge			X	X															
Follow-up ^5^					X		X		X		X		X		X		X		X
Clinical review					X		X		X		X								X
Pill counts ^6^					X		X		X		X								X
Liver function tests ^7^					X		X		X		X								X
Safety monitoring blood tests ^8^					X		X		X		X								X
PK sampling ^9^							X	X	X										
1HP initiation (late outpatient arm)							X												
ART initiation / switch ^10^					X	X	X												
Primary outcome ^11^																			X
Trial completion																			X

1. Participants will receive antifungal for 1–2 weeks as induction therapy for cryptococcal meningitis. Following completion of induction therapy, patients will step down to 800mg Fluconazole (continuation phase) to complete total 10-week course. 2. Clinical samples will be stored for future research 3. In cases, where the participant remains an inpatient for ≥14 days, randomisation will occur on day 14 rather than on the planned day of discharge. 4. 1HP = Rifapentine 600mg daily plus Isoniazid 300mg daily (plus pyridoxine) 5. Follow-up will occur on alternate weeks until week-18. 6. Self-reported adherence will be assessed, and pill counts conducted. 7. Alternate week liver function tests (LFTs) will be performed to screen for drug-induced liver injury for the duration of 1HP. 8. Safety monitoring blood tests will be taken including alternate week full blood count and renal function blood tests for the duration of 1HP. Blood will also be stored for future research studies. 9. Rifapentine / fluconazole / dolutegravir PK sampling will be conducted for 15 participants. 10. We anticipate that ~1/3 of participants will be ART naïve, these patients will start ART at week 4–6, ~1/3 of participants will be on ART but have clinical/immunological/virological failure, these patients will switch ART at week 4–6. Patients newly started on ART (<3-months prior to their cryptococcosis diagnosis) i.e. those with unmasking cryptococcal-IRIS will continue their ART. 11. Treatment completion is defined as participant reported adherence to >90% of the study medications, to be completed within 6-weeks of treatment initiation. TB-disease free is defined as not receiving a diagnosis of active TB disease for the duration of the trial.

***Primary intervention:*** Participants will be randomised to receive either a 28-day course of rifapentine 600mg daily plus isoniazid 300mg daily (1HP) to be initiated as an inpatient or as an outpatient. Amongst participants in the inpatient initiation arm, 1HP TPT will be started in hospital during the second week after cryptococcal meningitis diagnosis. Amongst participants in the outpatient initiation arm, 1HP TPT will be started in the outpatient clinic during the sixth week after cryptococcal meningitis diagnosis. The initial HP treatment dose will be given as directly observed therapy (DOT) in a healthcare setting for both intervention arms (either in the hospital or in the outpatient clinic), thereafter 1HP will be self-administered. Adjunctive pyridoxine (25mg/day) will be provided for all study participants to reduce the risk of peripheral neuropathy.

***Inpatient management:*** Participants will have a full history and examination at time of cryptococcal meningitis diagnosis and will be reviewed daily whilst admitted. Following completion of induction anti-fungal therapy for cryptococcal meningitis, participants may be discharged at the discretion of the attending study physician. Hospital discharge will typically occur ~7–14 days following cryptococcal meningitis diagnosis and commencement of anti-fungal therapy. Amongst participants in the inpatient initiation 1HP arm at least the first dose of 1HP must be given as in inpatient. Following hospital discharge participants will be followed-up every two weeks until week-18.

***Outpatient management:*** This will include: (1) clinical review with TB symptom screen and full physical examination; (2) 1HP adherence review with pill counts during 1HP receipt; (3) safety monitoring blood tests for the duration of 1HP; (4) additional myco-bacteriological and/or radiological testing for active TB disease at the discretion of the study physician as clinically indicated; (5) ART planning and counselling. Participants randomised to the outpatient initiation arm will commence 1HP during week 6.

***Assessment of adherence:*** Adherence to 1HP treatment will be assessed by means of participant interview and pill counts at follow up visits. 1HP treatment completion will be defined as participant reported adherence to >90% of the study medications, to be completed within 6-weeks from treatment initiation. Discontinuation will be defined as cessation of the study drugs for ≥ 5 consecutive days for any reason.

***Blood test monitoring:*** Blood will be drawn prior to enrolment including liver function tests and hepatitis B surface antigen testing (HbsAg). Safety monitoring blood tests (liver function tests (LFTs), renal function tests, and full blood count) will thereafter be taken on alternate weeks for the duration of 1HP therapy **.** If a participant has abnormal LFTs (LFT above the upper limit of normal, which do not meet the exclusion criteria) at screening, outpatient follow-up with clinical review and safety monitoring blood tests will be weekly. Additional samples will be taken alongside monitoring blood tests for sub-studies, including PK/PD studies.

***Anti-retroviral therapy:*** HIV-positive participants who are ART naïve, or who have virological failure will initiate/switch ART at week 4–6 in line with WHO and Ugandan guidelines. Tenofovir, Lamivudine and Dolutegravir (TDF+3TC+DTG) will be the first line ART regimen as per WHO and Ugandan guidelines; there are no drug-drug interactions (DDIs) anticipated between isoniazid and TDF, 3TC or DTG, nor between rifapentine and TDF and 3TC. There is a potential DDI between rifapentine and DTG which will be evaluated in the PK sub-study. ART initiation/switch will be done in conjunction with the participant’s ART clinic with second-line or alternative regimens available as required.

### Treatment modifications, interruptions, and discontinuations

Study physicians may interrupt 1HP dosing at physician discretion for a potentially life-threatening adverse reaction. Study participants diagnosed with drug-induced liver injury will stop 1HP and it will not be recommenced. Study participants diagnosed with active TB disease during the study period will stop 1HP and commence treatment for active TB in line with drug-susceptibility testing. If a study participant becomes pregnant whilst receiving 1HP, 1HP will be discontinued and isoniazid preventative therapy (IPT) will be started in line with Ugandan guidelines.

Study participants who are randomised to outpatient initiation 1HP who are diagnosed with active TB disease, become pregnant, or who commence a protease inhibitor prior to week-6, will not initiate 1HP TPT as planned.

### Termination of study

Reasons for study termination are study completion (week 18), withdrawal of consent, death, or lost to follow up. At study termination the study follow-up and termination case report forms (CRF) will be completed documenting interval history, vital status, primary and secondary endpoints, and reason for study termination.

### Timeline

A total of 205 patients will be recruited over a period of 3 years. This is feasible based upon previous experience and rates of trial recruitment at our three clinical sites. IMPROVE study recruitment will be supported by regular sensitisation activities to facilitate referrals where appropriate from surrounding clinics and hospitals.

### Statistical methods

The primary endpoint, TB-disease free 1HP treatment completion is a composite measure of safety and feasibility. The primary endpoint will be analysed using a generalised linear model (GLM), with a binomial distribution and an identity-link function, from which the unadjusted risk difference between the treatment groups and its one-sided 95% CI will be presented. If the upper limit of the one-sided 95% CI falls below the non-inferiority margin of 15%, non-inferiority will be declared. Assuming an 80% TB-disease free completion rate in the outpatient initiation 1HP TPT arm, a sample size of 205 will give 80% power, with a one sided 95% confidence interval, to determine whether inpatient initiation of 1HP led to non-inferior TB-disease free completion rates at a 15% non-inferiority margin, allowing for an 5% rate of loss to follow-up and 10% post-randomisation mortality at 18-weeks.

Secondary endpoints including survival will be compared at trial completion (18-weeks after cryptococcal meningitis diagnosis and commencement of anti-fungal therapy) based on superiority test using a 5% two-sided significance level. Analysis of binary secondary outcomes will be conducted using logistic regression models to calculate the OR and two sided 95% CIs between the treatment groups. Analyses of survival data and incident TB will be conducted using unadjusted Cox regression analysis to calculate the HR and 95% CI between the treatment groups. Unadjusted models are appropriate in the context of randomisation, and given the potential for data sparsity. Kaplan–Meier survival curves from point of cryptococcal meningitis diagnosis through 18-weeks by TPT group will be calculated and displayed.

The safety analysis will be descriptive and the frequency and proportions of participants suffering clinical and laboratory-defined grade ≥3 AEs and SAEs will be generated by treatment arms. The safety analysis will include every participant who received a dose of HP.

### Ancillary studies

**1.**  **Drug-drug interactions caused by rifapentine associated CYP450 enzyme induction: implications for clinical management in cryptococcal meningitis and advanced HIV disease.**
**Study population:** 15 study participants with HIV-associated cryptococcal meningitis taking 1HP, fluconazole and dolutegravir.
**Hypothesis:** Our hypothesis is that neither fluconazole nor dolutegravir will require dose adjustment when co-administered with rifapentine, and therefore a standardized package of care including TPT can be provided for adults with HIV-associated cryptococcal meningitis without need for dose modification.
**Schedule of events:** PK sampling will be performed on day 0, 5 and 14 of 1HP therapy. Rifapentine, fluconazole, dolutegravir concentrations will be measured at five time-points on each PK-day using liquid chromatography-tandem mass spectrometry approach.
**Analysis:** Non compartmental analysis (Cmax, Cmin, AUC)

**Quality control and assurance**

Trial oversight will be provided by the trial monitoring group (TMG), trial steering committee (TSC) and an independent data safety and monitoring board (DSMB). The DSMB consists of three independent members: DSMB chair, DSMB statistician, and DSMB clinician; the role of the DSMB is to safeguard the interests of trial participants, monitor the main outcome measures including safety and efficacy, and monitor the overall conduct of the trial. The study sponsor is the London School of Hygiene and Tropical Medicine (LSHTM). LSHTM, Keppel Street, London, WC1E 7HT as the trial sponsor had no role in the trial design, and will not be involved in the collection, analysis, and interpretation of data; in the writing of the report; or in the decision to submit the paper for publication.

The sites will be monitored at regular intervals with visits by the principal investigator (PI) and the study monitor in order to monitor the conduct of the trial and ensure that the principles of International Conference of Harmonisation (ICH) Good Clinical Practice (GCP) are being adhered to. Recruiting sites will be visited by the study monitor and the PI at the site initiation visit (SIV) prior to recruitment commencement, after the first 10 participants, when 50% of recruitment is complete, and at trial closure. Additional visits will be conducted if required. Monitoring visits will ensure that all training has been completed, that drug supply and equipment are in place and that all staff are up to date on the protocol and procedures.

Central monitoring will be performed in addition to the on-site monitoring procedures. All grade ≥3 AEs, SAEs and Suspected Unexpected Serious Adverse Reactions (SUSARs) will be reported to the TMG within 24hrs; SAEs and SUSARs must also be reported to the local ethics and regulatory bodies (Mulago Hospital Institutional Review Board and Uganda National Council for Science and Technology) no later than 7 days after the investigators are first aware. Quarterly reports on the progress of the trial, as well as the frequency of The Division of AIDS (DAIDS) grade ≥3 AEs, SAEs and all SUSARs will be compiled by the PI/statistician and reviewed by the TMG and the local ethics and regulatory bodies. These reports will be compiled and presented to the TSC and the DSMB at least once every 6 months. Annual summative reports will be sent to the sponsor for review. The DSMB will also review participants’ safety data and frequency and causes of death. Any significant issues/protocol violations/serious breaches will also be communicated to the study monitor, sponsor, and Institutional review board (IRB).

### Data collection and management

Study source documents will include CRFs, laboratory results, radiology results and other relevant documents. Data entry will occur via the DataFax system: paper-based CRFs are scanned in by the study team, emailed to a remote server, and participant data is then entered by intelligent character recognition. After an initial automated error-checking, a second review for accuracy is performed by the DataFax team at the Infectious Diseases Institute, Uganda. The DataFax system allows for automated data queries to highlight any missing data in real time. DataFax also allows for remote review by oversight bodies and permanent archiving. CRFs will be harmonized between all study sites enabling multi-site data management. Essential source documents will be retained for 20-years after the completion of the study, as per Ugandan guidelines.

## Ethical considerations

***Patient confidentiality:*** All participant-related information (including CRFs, laboratory specimens, reports, etc.) will be kept strictly confidential. Participants will be identified only by means of a coded number specific to each participant. All computerised databases will identify participants by numeric codes only, and will be password-protected. All paper records will be kept in a secure, locked location and only research staff will have access to the records. HIV clinic records will be kept in the local HIV clinic as per local practice.

***Sample use and storage:*** Consent forms also include consent for storage of samples (blood and urine) in accordance with Uganda National Council for Science & Technology guidelines and LSHTM Human Tissue Act Policy. Participant specimens will be stored for current and future research studies related to opportunistic infections and the immune response in the IDI translational laboratory in accordance with local standards and LSHTM Human Tissue Act Policy.

***Data sharing with third parties:*** Upon request, participant records will be made available to the following named parties only: study sponsor, the sponsor’s monitoring representative, and applicable regulatory entities, including the Uganda National Council of Science and Technology and Mulago IRB. The anonymised database/protocol will be shared with the journal, if required.

***Ethical approval:*** The investigators have obtained approval from the Research Ethics Committees of the London School of Hygiene & Tropical Medicine (Ref: 24059, approved 07 June 2021), as well as Mulago Hospital IRB (Ref: MHREC 2021-25, approved 28 July 2021), and the Uganda National Council of Science and Technology (UNCST, Ref: HS1607ES, approved 25 August 2021). Any further amendments will be submitted and approved by each ethics committee, and communicated with all study investigators prior to implementation.

## Indemnity

The sponsor of the trial is the London School of Hygiene and Tropical Medicine and as such provides indemnity for the trial. All personnel involved in the trial will be expected to be indemnified by their employing authority.

## Publication policy

We will share results though presentations at scientific conferences and in peer-reviewed open-access journals.

## Study status

The first IMPROVE study participant was recruited on 21 January 2021. The IMPROVE study is actively recruiting.

## Discussion

The IMPROVE trial will provide preliminary safety and feasibility data to inform 1HP TPT strategies for adults with advanced HIV disease and cryptococcal meningitis. These data will be used to inform design of a subsequent phase 3 trial to evaluate the efficacy of inpatient 1HP TPT in preventing TB disease and deaths in AHD. The potential impact of demonstrating that inpatient initiation of 1HP TPT is safe and acceptable for this high-risk AHD subpopulation, would be to expand the range of clinical encounters in which clinicians can feasibly provide TPT for PLHIV. Data suggest that amongst PLHIV, of those offered TPT more that 90% agree to start, and reported treatment completion for short course rifapentine-containing TPT regimens (1HP/3HP) is >90% ^13, 14^; the vast majority of losses from the LTBI care cascade therefore occur up-stream and are driven by health system factors rather than patients. Collapsing the LTBI care cascade during hospitalisation to enable rapid TPT initiation prior to discharge, could significantly increase the proportion of adults with AHD successfully initiated on TPT, and therefore reduce the incidence of active TB disease and TB-associated deaths within this key population. 

## Data Availability

No underlying data are associated with this article. Zenodo: Integrated management of cryptococcal meningitis and concurrent opportunistic infections to improve outcomes in advanced HIV disease: a randomised strategy trial. https://doi.org/10.5281/zenodo.7858543 ^21^. This project contains the following extended data: IMPROVE 1HP RCT PIS - English V6.0 November 2022.docx IMPROVE consent form - English V2 3rd March 2022.docx Zenodo: SPIRIT checklist for ‘Integrated management of cryptococcal meningitis and concurrent opportunistic infections to improve outcomes in advanced HIV disease: a randomised strategy trial’. https://doi.org/10.5281/zenodo.7858543 ^21^. Data are available under the terms of the Creative Commons Attribution 4.0 International license (CC-BY 4.0).
