# Supplementary material for: Integrated management of cryptococcal meningitis and concurrent opportunistic infections to improve outcomes in advanced HIV disease: a randomised strategy trial
Source: Wellcome Open Res. 2024 Jun 5;9:14. Originally published 2024 Jan 8. [Version 2] doi: 10.12688/wellcomeopenres.19324.2 (PMC11157187; doi:10.12688/wellcomeopenres.19324.2)
Supplement: Peer review history

# Reviewer report: Reviewer response for version 2

**DOI:** 10.21956/wellcomeopenres.24870.r85832

**Published:** 2025-04-09

**Authors:** Ajay Rangaraj

**Affiliations:** 1 Department of HIV, Hepatitis and STIs, World Health Organisation, Geneva, Switzerland

Just had some comments in relation to the different amphotericin formulations as ADR will likely be different - just to say there has to be some sort of sub-analysis between those who receive treatment. 

 Otherwise no other comments - thanks for the revisions and the detailed responses.

Is the study design appropriate for the research question?

Yes

Is the rationale for, and objectives of, the study clearly described?

Yes

Are sufficient details of the methods provided to allow replication by others?

Yes

Are the datasets clearly presented in a useable and accessible format?

Not applicable

Reviewer Expertise:

I work on synthesising evidence for and coordinating global treatment guidelines updates for HIV treatment and care - particularly ART, Advanced HIV disease, cryptococcal meningitis, histoplasmosis and other opportunistic infections.

I confirm that I have read this submission and believe that I have an appropriate level of expertise to confirm that it is of an acceptable scientific standard.

# Reviewer report: Reviewer response for version 2

**DOI:** 10.21956/wellcomeopenres.24870.r85833

**Published:** 2024-06-06

**Authors:** Vincent Ganu

**Affiliations:** 1 Korle-Bu Teaching Hospital, Accra, Ghana

I have reviewed the authors' responses to my review comments and also seen their revised manuscript. The responses are well noted and I ask that we ensure the responses are reflected in the revised manuscript as some are not reflecting in the manuscript. Aside from that, it can be approved and accepted. Thank you.

Is the study design appropriate for the research question?

Yes

Is the rationale for, and objectives of, the study clearly described?

Partly

Are sufficient details of the methods provided to allow replication by others?

Partly

Are the datasets clearly presented in a useable and accessible format?

Not applicable

Reviewer Expertise:

NA

I confirm that I have read this submission and believe that I have an appropriate level of expertise to confirm that it is of an acceptable scientific standard.

# Reviewer report: Reviewer response for version 1

**DOI:** 10.21956/wellcomeopenres.21407.r78401

**Published:** 2024-05-02

**Authors:** Sonal Munsiff

**Affiliations:** 1 University of Rochester Medical Center: Strong Memorial Hospital, Rochester, NY, USA

The main objective of this study which is going to be an open label, two arm randomized controlled trial, is to assess the safety and feasibility  of giving 1 month of isoniazid and rifapentine regimen for TB preventive therapy in adults with recently diagnosed HIV associated cryptococcal meningitis.  The objective is laudable and the study worth doing.

 Overall the protocol is well written but there are several areas where I think some more detail would be helpful to the reader.  In addition, there is a serious flaw in rationale for using the current regimen in light of known sever DDI between dolutegravir and rifapentine, as noted below.

 In the introduction, paragraph 4, page 3, the authors state that ultra short course TPT may have particular advantages over longer courses. They state that one clear benefit is respect to Drug drug interactions. However, most of the ART regimens now in use have significant interaction with rifapentine and therefore correct dosing of dolutegravir and rifapentine remains an ongoing challenge.

 Further on in that same paragraph they state that “the risk of unmasking TB-IRIS, however, remains following ART initiation at 4-6 weeks, with most incident IRIS  events occurring within the first month of ART initiation.”  It would be helpful here for the reader to have some data on the risk of TB IRIS in the population being studied, rather than just citing referral references.

 Also, the authors state further in that paragraph, that “Completion of 1HP TPT prior to ART initiation has the potential to reduce incidence of TB-IRIS, active TB disease, and TB deaths among this subpopulation.” Again, data on the risk of these events would be helpful.

 In the section on Anti-retroviral therapy on page 7 they state that “there is a potential DDI between  rifapentine and DTG, which will be evaluated in the PK sub-study.”  In fact, there is a very well known interaction between DTG and rifapentine, and numerous studies have shown this, and this is not addressed at all in this protocol. This known DDI should all be addressed.  

 There is serious concern that using standard dose dolutegravir with rifapentine can lower dolutegravir levels enough to lead to integrase resistance.   The current recommendation by WHO is to use double dose DTG (50 milligrams twice a day instead of 50 milligrams once a day), when given with rifapentine 1 HP.   

 The only way to know that DTG dose is adequate is by looking at outcomes of HIV viral loads, along with the PK data.  The schedule of events that is given does not look at HIV viral load at any time point. Also, an N of 15 for the PK sub study is likely inadequate. 

 The hypothesis (page 8) that neither fluconazole nor dolutegravir will require dose adjustment when co-administered with rifapentine is therefore not based on the available data. 

 The authors may want to consider that a good rationale for giving early TB preventive therapy with 1HP regimen is that it could be completed before anti-retroviral therapy is started in ART nauve people with cryptococcal meningitis, at 6 weeks after meningitis treatment, so that drug drug interactions between ART and rifapentine could be avoided.

 Another thing to remember is that DTG levels could be affected even after rifapentine is stopped as the effect of rifapentine on the cytochrome P450 system is expected to persist for one to two weeks after stopping the rifapentine. In general, for people on DTG who get 1HP regimen, it is recommended to continue double dose DTG for 2 weeks after stopping rifapentine.

Is the study design appropriate for the research question?

No

Is the rationale for, and objectives of, the study clearly described?

Partly

Are sufficient details of the methods provided to allow replication by others?

Yes

Are the datasets clearly presented in a useable and accessible format?

Not applicable

Reviewer Expertise:

Tuberculosis treatment Clinical trials research, tuberculosis program evalaution, HIV and TB co-treatment challenges, HIV Clinical trials

I confirm that I have read this submission and believe that I have an appropriate level of expertise to confirm that it is of an acceptable scientific standard, however I have significant reservations, as outlined above.

# Response

**Published:** 2024-07-30

**Authors:** Jayne Ellis

**Affiliations:** Infectious Diseases Institute, Uganda

Dear Sonal Munsiff,

 Thank you for taking the time to review the IMPROVE protocol - your feedback is very much appreciated. Please find below responses to each of your comments raised:

**The main objective of this study which is going to be an open label, two arm randomized controlled trial, is to assess the safety and feasibility  of giving 1 month of isoniazid and rifapentine regimen for TB preventive therapy in adults with recently diagnosed HIV associated cryptococcal meningitis.  The objective is laudable and the study worth doing.**

**Overall the protocol is well written but there are several areas where I think some more detail would be helpful to the reader.  In addition, there is a serious flaw in rationale for using the current regimen in light of known sever DDI between dolutegravir and rifapentine, as noted below.** **In the introduction, paragraph 4, page 3, the authors state that ultra short course TPT may have particular advantages over longer courses. They state that one clear benefit is respect to Drug drug interactions. However, most of the ART regimens now in use have significant interaction with rifapentine and therefore correct dosing of dolutegravir and rifapentine remains an ongoing challenge.** **Further on in that same paragraph they state that “the risk of unmasking TB-IRIS, however, remains following ART initiation at 4-6 weeks, with most incident IRIS events occurring within the first month of ART initiation.” It would be helpful here for the reader to have some data on the risk of TB IRIS in the population being studied, rather than just citing referral references.**

**Response:** Thank you for this feedback. There are no published data on the incidence of TB IRIS specifically amongst adults with HIV-associated cryptococcal meningitis. The two chosen citations referenced within the protocol are taken from comparably immunocompromised cohorts, in comparable settings. In Haddow’s (2012) cohort of 498 adults initiating ART in South Africa, 22.9% of participants developed IRIS (36% paradoxical, 64% unmasking), with TB disease accounting for 25% of all IRIS events. Within this study 71.2% of IRIS events occurred within the first 6 after ART initiation. Comparably, in Worodria’s (2012) study of 376 participants initiating ART in Kampala Uganda, a total of 53 (21%) patients developed TB-IRIS, at a median of 2 weeks (IQR 12–22 days) after starting ART. We feel these are suitable references to demonstrate that most TB-IRIS events occurs soon after ART initiation. Furthermore, we anticipate that within our current context of DTG-based ART, the majority of IRIS events will in fact occur even earlier than as described in these two cohort studies from the pre-integrase inhibitor era.

**Also, the authors state further in that paragraph, that “Completion of 1HP TPT prior to ART initiation has the potential to reduce incidence of TB-IRIS, active TB disease, and TB deaths among this subpopulation.” Again, data on the risk of these events would be helpful.**

**Response:** Thank you for this feedback. The chosen citation for this statement comes from the REALITY trial. The REALITY trial investigated the use of enhanced anti-microbial prophylaxis including 12 weeks of isoniazid–pyridoxine TB preventive therapy (TPT) and ART, as compared to standard prophylaxis to improve treatment outcomes amongst ART naïve adults and children with a baseline CD4 count <100 cells/mm ^3^. Alongside improved 24-week survival, the REALITY trial demonstrated that enhanced-prophylaxis including TPT significantly reduced IRIS-events including TB-IRIS events. TB-IRIS events occurred in 4.2% the enhanced-prophylaxis arm vs. 7.7% in the standard-prophylaxis arm (p=0.002). Based on the REALITY trial and IRIS literature as referenced, we anticipate the incidence of TB-IRIS to be low (~5%) within our clinical trial participants receiving 1HP TPT. We have added his estimate into the updated version of the protocol. We hypothesise that incident TB diagnoses (including unmasking TB-IRIS) may be less frequent amongst participants initiated on 1HP TPT prior to hospital discharge, with completion prior to ART initiation/switch at week 4-6 as compared to those who initiate 1HP TPT in the outpatient setting at week-6.

**In the section on Anti-retroviral therapy on page 7 they state that “there is a potential DDI between rifapentine and DTG, which will be evaluated in the PK sub-study.”  In fact, there is a very well known interaction between DTG and rifapentine, and numerous studies have shown this, and this is not addressed at all in this protocol. This known DDI should all be addressed.**

**Response:** Thank you for this feedback. As stated in the protocol we recognise that rifapentine as contained in 1HP induces certain cytochrome P-450 enzymes which therein increases the metabolism of dolutegravir (DTG); this indeed is a known drug-drug interaction (DDI). What is not known is whether this DDI is clinically significant and whether dose adjustment is required. It is for this reason we are conducting the IMPROVE pharmacokinetic (PK) sub-study. We have added “clinically significant” to the updated version of the protocol for clarity. It is not known whether DTG dose modification is required with co-administration of 1HP. Whilst certainly HP and DTG should be co-prescribed with caution due to the risk as described in the protocol, due to a lack of data, currently, neither the World Health Organization (WHO) nor the Ugandan Ministry of Health, have made any specific dosing recommendations regarding the co-administration with DTG and 1HP, and do not currently recommend double dosing of DTG. Rather due to a lack of definitive data, the WHO guideline development group stressed the continued need for more PK studies, as we are performing, to investigate the  concurrent use of 1HP and ART. Current WHO guidelines were informed by data as presented at CROI 2019 and later published by Dooley *et al.* (2020), from the DOLPHIN trial – a PK study investigating the co-administration of 3HP and DTG amongst people living with HIV in South Africa. Participants received 50 mg of daily DTG for 8 weeks, then began once-weekly rifapentine (900 mg)-isoniazid (900 mg) for 12 weeks. The DOLPHIN trial demonstrated that although co-administration with 3HP decreased DTG bioavailability, which was associated with a modest decrease in DTG trough levels, all trough levels but one were above the DTG IC90 (90% maximal inhibitory concentration). Furthermore, within the DOLPHIN study, all HIV viral loads as measured at baseline, 11-weeks and 24-weeks were suppressed (defined as < 40 copies/mL) in all study participants. Therefore the authors concluded that DTG may be co-administered with 3HP *without* dose adjustment. To date, there have been no PK studies to date investigating the co-administration of 1HP and DTG. *There is currently clinical equipoise as to whether once or twice daily dolutegravir should be used*; to note, a large multi-country ACTG trial is planned which will compare dosing strategies and provide the definitive PK data on 1HP and DTG co-administration. Cognizant that CY450 enzyme induction takes ~2 weeks from treatment initiation, it is possible that given the shorter duration of 1HP vs. 3HP (one month of daily rifapentine therapy, as opposed to 3-months of weekly rifapentine therapy), 1HP will in fact result in a more modest decrease in DTG bioavailability and DTG trough levels. As a result of these considerations:

1. that there is currently no evidence base to guide DTG dosing in the context of 1HP, with clinical equipoise regarding once or twice daily dosing, indicated by the fact that a large ACTG trial is currently investigating this question;
2. that the WHO and Ugandan national guidelines do not recommend double DTG dosing in this context; and
3. that data using 3HP suggests that once daily DTG dosing is adequate,

We have opted to use once daily DTG dosing with careful monitoring. We acknowledge that, given the clinical equipoise, it would have been equally valid to opt for twice daily dosing. With the current protocol, the IMPROVE PK study will provide preliminary data that will support the results of the DOLPHIN study, and may help demonstrate that HP can be co-administered with DTG without dose adjustment, which would be extremely beneficial from a programmatic perspective.

**There is serious concern that using standard dose dolutegravir with rifapentine can lower dolutegravir levels enough to lead to integrase resistance. The current recommendation by WHO is to use double dose DTG (50 milligrams twice a day instead of 50 milligrams once a day), when given with rifapentine 1 HP.** 

**Response:** WHO guidelines suggest that DTG needs to be increased to 50 mg twice daily when given together with rifampicin. As explained above, due to a lack of data, the WHO have not made any specific dosing recommendations regarding the co-administration with DTG and 1HP.

**The only way to know that DTG dose is adequate is by looking at outcomes of HIV viral loads, along with the PK data. The schedule of events that is given does not look at HIV viral load at any time point.**

**Response:** Thank you for making this important point. To clarify, HIV viral load testing is performed as part of clinical care, and within our longitudinal cohort study of adults with advanced HIV disease and suspected meningitis. We are collating these HIV VL results and we be able to characterise virological suppression amongst IMPROVE participants, these data will be presented as part of the PK analyses.

**Also, an N of 15 for the PK sub study is likely inadequate.**

**Response:** The IMPROVE PK sub-study – including power considerations - was been designed with the input of leading PK experts. We recognise that a larger study would be preferable but this is outside the scope of the IMPROVE trial. As noted above, the forthcoming large multi-country ACTG will provide the definitive PK data on 1HP and DTG co-administration.

**The hypothesis (page 8) that neither fluconazole nor dolutegravir will require dose adjustment when co-administered with rifapentine is therefore not based on the available data.**

**Response:** This hypothesis regarding 1HP dosing, is guided by results from the DOLPHIN study with further justification as described above. The hypothesis that fluconazole does not require dose adjustment in the context of 1HP is based on the available PK data using rifamycins, and expert opinion. Rifampicin administration does reduce fluconazole levels, however this is not of clinical significance in terms of treatment outcomes, and in the context of very high fluconazole dosing used in cryptococcal meningitis induction and consolidation treatment (800 – 1200mg/day) the consensus view is that adequate fluconazole levels will be obtained, and toxicity risks with and further fluconazole dose increases would outweigh any benefits, particularly given the dual risks of hepatotoxicity.

**The authors may want to consider that a good rationale for giving early TB preventive therapy with 1HP regimen is that it could be completed before anti-retroviral therapy is started in ART naive people with cryptococcal meningitis, at 6 weeks after meningitis treatment, so that drug drug interactions between ART and rifapentine could be avoided.**

**Response:** Thank you, we recognise that this would be a specific benefit for those presenting to care who are ART naïve.

**Another thing to remember is that DTG levels could be affected even after rifapentine is stopped as the effect of rifapentine on the cytochrome P450 system is expected to persist for one to two weeks after stopping the rifapentine. In general, for people on DTG who get 1HP regimen, it is recommended to continue double dose DTG for 2 weeks after stopping rifapentine.**

**Response:** We do not plan to conduct any PK testing beyond 1HP completion, as this is beyond the scope of the IMPROVE PK sub-study. Whilst the IMPROVE PK sub-study will provide useful preliminary data, as noted above, the forthcoming large multi-country ACTG will provide the definitive PK data on 1HP and DTG co-administration. Thank you again for your review.

 Very best wishes,

 Dr Jayne Ellis

# Reviewer report: Reviewer response for version 1

**DOI:** 10.21956/wellcomeopenres.21407.r78395

**Published:** 2024-04-28

**Authors:** Vincent Ganu

**Affiliations:** 1 Korle-Bu Teaching Hospital, Accra, Ghana

**Title:**

 In the protocol, the opportunistic infection of interest is TB but what the authors are interested in is TPT. And so I suggest we state it as such (i.e. Integrated management of crypto and concurrent TB preventive therapy) in the title rather than stating opportunistic infections which is very broad.

**Abstract:**

 “IMPROVE” should be written in full as it is the first time of mention.

**Methods:**

 Will there be use of standardized questionnaire to collect information about participants? If so, lets state and state the variables that will be collected with the questionnaire

**Figure 1:**

 How did we get the 450 and the 205? And how are the 205 selected from the 450?

 DILI should be added to the abbreviation lists and written in full.

**Primary Objective:**

 I thought the aim of the study was to compare the safety and feasibility of 1 HP among 2 groups (i.e. In-patient and outpatient) to establish non-inferiority of both strategies or approaches hence the study design chosen (trial with randomization).

 Because for the primary objective currently stated, the current study design is not needed to answer this question. Could have done a cohort study (prospective or retrospective) to answer the primary objective as it is stated now. Need to rephrase to align with the study design/approach.

**Study setting:**

 Kindly give additional information about study sites to demonstrate they have the existing case load of cryptococcal meningitis for the conduct of this study within the study time period as the prevalence of crypto is low among HIV patients. A little more information to put things in perspective will be appreciated. A fair idea of the HIV patient numbers, admission figures and mortality/survival figures relating to each of these facilities.

**Primary endpoint:**

 Need to be specific on the starting time point of calculating the 18 weeks. Is it after diagnosis of crypto or commencement of antifungal therapy as the two may not necessarily occur on the same day due to implementation challenges.

 This definition for “treatment completion “does not fit there but rather fits after point 1 under secondary endpoints.

**Inclusion criteria:**

 How do the investigators plan on ensuring that the patients are HIV positive. They should state the steps they will take to ensure this (the tests to be done and the type of tests to be used) so that any other researcher can follow and do same if conducting study elsewhere.

**Exclusion criteria:**

 I think a *major* activity in this study will be the screening of potential participants for TB to rule out active TB.

1. The authors should have a sub-section titled study procedure under which they detail all their activities. One of the main activities to detail will be how the potential participants will be screened for TB.
2. In the flow diagram, several tests have been stated for TB screening. Will each participant be screened using all the tests? If no, who receives what and what is the justification for each test for a participant. The urine LAM is usually best for patients with CD4 cell count <100 or 200. So who gets to do this? GeneXpert will screen sputum. Who gets to do this test and who does not? Is very participant receiving an x-ray? Who gets to do it and who does not?
3. Because this is a study, how will active TB be diagnosed? It must be stated in full as there are different test being proposed to rule out active TB.
4. And who does the TB diagnosis to ensure agreement on what is TB and what is not TB? It is best if there are 2 or more experts reviewing the tests outcomes to agree on what is considered active TB and what is not.
5. What about sputum negative TB diagnosis as occurs in clinical practice? Are they excluded from the study as well?
6. For standardization purposes, where will the various TB tests be done and the procedure for each test should be clearly outlined. This is to ensure that the study is reproducible if other researchers want to pursue same study in other jurisdictions.
7. As part of the patient screening, will each patient do liver function test, hepatitis B surface antigen? These must be clearly stated as part of study activities.
8. Based on the above, then it means there will be a screening phase for the study so patient’s samples are taken for analysis. Then based on the outcomes of the test results, there will be the enrolment phase where those eligible will be included in the study. I think these should be clearly outlined under the study procedure in a systematic way that every reader can follow

**Withdrawals:**

 I think if the patient says they have withdrawn from the study, then there is no need to do any follow up. There is nothing like partially withdrawn or incomplete withdrawal.

**Randomization or treatment allocation:**

**“In instances, where the participant remains an inpatient for ≥14 days”.**

 This should be clarified as it will have an impact on randomization and the patient’s treatment outcomes. Is it 14 days from initial admission? Or 14 days after diagnosis of crypto meningitis? Or 14 days after start of treatment for crypto meningitis? This is important because there will be a time lag between patient presenting to the hospital, carrying out a lumbar puncture procedure and then a turn-around time for getting results of CSF analysis. And patient is still on admission when all these are being done.

**Table 1:**

- What antifungals are being used? Are these antifungals be administered via intravenous route or via oral route or a mixture? Will it be standard for all patients? Is the study providing these antifungals for the patients as part of the study for standardization purpose? It’s important to clarify the above as it will have an impact on patient treatment outcomes and be a confounder of the study. Reason why all the above needs to be taken into consideration and stated in the protocol.
- What will the pill count be assessing? Fluconazole or 1HP or both or drugs for other co-morbidities? These need to be stated clearly. Assessment of adherence to all medications is key as it has an effect or impact on treatment outcomes and may be confounders. We are looking at integration of management. Another reason why we should monitor for adherence on all medications.
- What drug will the patient be taking after completing the 10 week continuation phase? This should be stated in the study procedure sub-section as the study follow up period is for 18 weeks.

“ **We anticipate that ~1/3 of participants will be ART naïve, these patients will start ART at week 4–6, ~1/3 of participants will be on ART but have clinical/immunological/virological failure**”

 What are these anticipations based on? What is the evidence for this or is it an educated guess? Is it from literature or from figures at the study sites? Too much has been summarized in this tables and needs to be explained under the methods section above

**Inpatient management:**

 “ **Following hospital discharge participants will be followed-up every two weeks until week-18”**

 What measures are the authors putting in place to reduce lost to follow up during the follow-up period? All these should be stated clearly? Are there incentives such as tokens for transport purposes for patients to increase their chances of coming for review? Are information on their phone contacts and those of caregivers being taken to do follow-up? Who will be one doing the follow-up? All these should be clearly outlined in the protocol

**Outpatient management (point 4):**

 The study physician is part of the research group and there must be a guideline or protocol to be followed for ruling out active TB. This should be stated. It cannot be at the discretion of the study physician as it affects the rigorousness of the study

**Sample size:**

- What is the justification for this sample size? How was it calculated and what were the basis for the calculation?
- Since there are 2 groups, is it 205 per group OR the 2 groups make up the 205?
- Will be good to state the formula used or the software used and the reference for readers to follow. And this should not be mixed with the statistical analysis methods
- What is the basis for selection of 5% as the loss to follow up rate and not 10% or 15%?

Is the study design appropriate for the research question?

Yes

Is the rationale for, and objectives of, the study clearly described?

Partly

Are sufficient details of the methods provided to allow replication by others?

Partly

Are the datasets clearly presented in a useable and accessible format?

Not applicable

Reviewer Expertise:

NA

I confirm that I have read this submission and believe that I have an appropriate level of expertise to confirm that it is of an acceptable scientific standard, however I have significant reservations, as outlined above.

# Response

**Published:** 2024-05-09

**Authors:** Jayne Ellis

**Affiliations:** Infectious Diseases Institute, Uganda

**Dear Vincent Ganu,** **Thank you for taking the time to review the IMPROVE protocol - your feedback is very much appreciated. Please find below responses to each of your comments raised:**

**Title: In the protocol, the opportunistic infection of interest is TB but what the authors are interested in is TPT. And so I suggest we state it as such (i.e. Integrated management of crypto and concurrent TB preventive therapy) in the title rather than stating opportunistic infections which is very broad.** Thank you for this feedback but we cannot change the IRB-approved study title at this stage.

**2. Abstract:**

**“IMPROVE” should be written in full as it is the first time of mention.** Thank you, this has been amended.

**3. Methods:**

**Will there be use of standardized questionnaire to collect information about participants? If so, lets state and state the variables that will be collected with the questionnaire** Thank you, the following has now been included “Case report forms (CRFs) detailing baseline demographics, clinical details including symptoms, clinical examination findings, ART history, TB and TPT history, and laboratory parameters will be collected.” **4. Figure 1:**

**How did we get the 450 and the 205? And how are the 205 selected from the 450?**

**DILI should be added to the abbreviation lists and written in full.** 450 adults with HIV-associated cryptococcal meningitis will have enhanced TB screening as part of the parent study. Only those with no evidence of active TB disease, will be eligible for consent and recruitment into the IMPROVE TB preventive therapy trial (n=205). A footnote has been added to the figure to explain this more clearly.

**5. Primary Objective:**

**I thought the aim of the study was to compare the safety and feasibility of 1 HP among 2 groups (i.e. In-patient and outpatient) to establish non-inferiority of both strategies or approaches hence the study design chosen (trial with randomization).**

**Because for the primary objective currently stated, the current study design is not needed to answer this question. Could have done a cohort study (prospective or retrospective) to answer the primary objective as it is stated now. Need to rephrase to align with the study design/approach.** Thank you, the primary objective has been amended to read: “To generate comparative data to compare the safety (adverse events) and feasibility (adherence and tolerability) of inpatient vs. outpatient initiation of 1HP TPT amongst adults with HIV-associated cryptococcal meningitis.”

**6. Study setting:**

**Kindly give additional information about study sites to demonstrate they have the existing case load of cryptococcal meningitis for the conduct of this study within the study time period as the prevalence of crypto is low among HIV patients. A little more information to put things in perspective will be appreciated. A fair idea of the HIV patient numbers, admission figures and mortality/survival figures relating to each of these facilities.** Our team have been conducting cryptococcal meningitis studies across these sites 2006, and these recruitment estimates were based on our trial experience in this setting. To give you some contemporaneous data, in the last 3-months, we have recruited 26 adults with HIV-associated cryptococcal meningitis at Mulago National Referral Hospital, 14 from Kiruddu National Referral Hospital, and 12 from Mbarara Regional Referral Hospital (i.e. ~17 per month in total across all sites).

**7. Primary endpoint:**

**Need to be specific on the starting time point of calculating the 18 weeks. Is it after diagnosis of crypto or commencement of antifungal therapy as the two may not necessarily occur on the same day due to implementation challenges.** Thank you for this feedback. This is 18-weeks from when the participant is recruited into the parent study, and we therefore refer to diagnosis of cryptococcal meningitis and commencement of anti-fungal within the study. We appreciate that if a patient was referred to our team with a suspected diagnosis of cryptococcal meningitis, there may be, as you describe a lag-period, however for study purposes we do not confirm the diagnosis of cryptococcal meningitis until study-team performed CSF CrAg has been confirmed as positive. Within the study, we always ensure that anti-fungal medications are started on the same day as the diagnosis is made. **This definition for “treatment completion “does not fit there but rather fits after point 1 under secondary endpoints.** 1HP adherence (defined as adherence to >90% of the 1HP study medications, to be completed within 6-weeks from treatment initiation) is a key component of the primary outcome, and key to assessing the feasibility of giving 1HP to this patient population. We have therefore left maintained this definition within the primary end-point heading, but have additionally included it as well alongside the secondary endpoints as suggested.  

**8. Inclusion criteria:**

**How do the investigators plan on ensuring that the patients are HIV positive. They should state the steps they will take to ensure this (the tests to be done and the type of tests to be used) so that any other researcher can follow and do same if conducting study elsewhere.** To be suitable for screening for the IMPROVE trial, a patient needs to be receiving treatment for HIV-associated cryptococcal meningitis i.e. the diagnosis of HIV and cryptococcal meningitis is performed by the parent cryptococcal meningitis study prior to IMPROVE screening. IMPROVE screening, consent and randomisation occurs just prior to hospital discharge. In terms of HIV screening within the parent study, where HIV status is unknown, counselling and HIV testing is performed using a fourth generation point-of-care test HIV antigen/antibody test. If a participant is known HIV-positive e.g if taking ART at admission, repeat HIV testing is not repeated.

**Exclusion criteria:**

**I think a *major* activity in this study will be the screening of potential participants for TB to rule out active TB. **

- **The authors should have a sub-section titled study procedure under which they detail all their activities. One of the main activities to detail will be how the potential participants will be screened for TB.**

Thank you, the following “eligibility screening” section has now been added to the manuscript: All potential study participant screened for the IMPROVE trial have the following TB diagnostics performed: urine Alere TB-LAM, urine Xpert Ultra, TB blood culture, and chest radiology. Additional laboratory (including Xpert Ultra on clinical samples (sputum / CSF / lymph node aspirate), and radiological tests may also be undertaken dependent on clinical presentation.

- **In the flow diagram, several tests have been stated for TB screening. Will each participant be screened using all the tests? If no, who receives what and what is the justification for each test for a participant. The urine LAM is usually best for patients with CD4 cell count <100 or 200. So who gets to do this? GeneXpert will screen sputum. Who gets to do this test and who does not? Is very participant receiving an x-ray? Who gets to do it and who does not?**

Yes, as per my paragraph above, all potential participants get urine Alere TB-LAM, urine Xpert Ultra, TB blood culture, chest radiology +/- additional TB diagnostics depending on the clinical presentation.  

- **Because this is a study, how will active TB be diagnosed? It must be stated in full as there are different test being proposed to rule out active TB.**

In terms of screening for IMPROVE eligibility, this is as stated in the exclusion criteria: “any patient with active TB disease (as evidenced by any positive TB screening test or taking TB therapy at time of screening) will not be eligible for enrolment.” i.e. a TB diagnosis may be microbiological (definite TB disease), or clinical TB disease (empirical treatment based on a clinical and/or radiological syndrome without a confirmed microbiological diagnosis).

- **And who does the TB diagnosis to ensure agreement on what is TB and what is not TB? It is best if there are 2 or more experts reviewing the tests outcomes to agree on what is considered active TB and what is not.**

Screening for IMPROVE eligibility is performed by the study team and any complex cases are discussed during a weekly team meeting. Any clinical or microbiological evidence of TB disease must be considered as an exclusion criteria. Only if the screening study doctors believe that the patient does not have active TB disease, should they be consider for inclusion into the trial.

- **What about sputum negative TB diagnosis as occurs in clinical practice? Are they excluded from the study as well?**

Yes clinical TB disease is also an exclusion criteria.

  

- **For standardization purposes, where will the various TB tests be done and the procedure for each test should be clearly outlined. This is to ensure that the study is reproducible if other researchers want to pursue same study in other jurisdictions.**

All TB tests ( are performed in accredited laboratories and/or using accredited tests following standard procedures and manufacturers guidelines.

  

- **As part of the patient screening, will each patient do liver function test, hepatitis B surface antigen? These must be clearly stated as part of study activities.**

This is as per current stated under “Blood test monitoring: Blood will be drawn prior to enrolment including liver function tests and hepatitis B surface antigen testing (HbsAg).”

  

- **Based on the above, then it means there will be a screening phase for the study so patient’s samples are taken for analysis. Then based on the outcomes of the test results, there will be the enrolment phase where those eligible will be included in the study. I think these should be clearly outlined under the study procedure in a systematic way that every reader can follow. **

Thank you, as per the above a section entitled eligibility screening has been added to the manuscript.

**Withdrawals:**

**I think if the patient says they have withdrawn from the study, then there is no need to do any follow up. There is nothing like partially withdrawn or incomplete withdrawal.**   Although these participants will not contribute to the primary end-point analysis, it will still be important to describe survival status amongst those withdrawn if consent is given.

**Randomization or treatment allocation:**

**“In instances, where the participant remains an inpatient for ≥14 days”.**

**This should be clarified as it will have an impact on randomization and the patient’s treatment outcomes. Is it 14 days from initial admission? Or 14 days after diagnosis of crypto meningitis? Or 14 days after start of treatment for crypto meningitis? This is important because there will be a time lag between patient presenting to the hospital, carrying out a lumbar puncture procedure and then a turn-around time for getting results of CSF analysis. And patient is still on admission when all these are being done.**   This is ≥14 days from study-confirmed diagnosis of cryptococcal meningitis and being recruited into the parent study.

**Table 1: **

- **What antifungals are being used? Are these antifungals be administered via intravenous route or via oral route or a mixture? Will it be standard for all patients? Is the study providing these antifungals for the patients as part of the study for standardization purpose? It’s important to clarify the above as it will have an impact on patient treatment outcomes and be a confounder of the study. Reason why all the above needs to be taken into consideration and stated in the protocol.**

All IMPROVE participants receive amphotericin-based induction anti-fungal therapy. The induction regimen received however, does depend upon drug availability and whether the participant received induction anti-fungal therapy as part of a randomized controlled trial. These data are collected, however given this is an RCT we expect all baseline co-variates including anti-fungal regimens to be well balanced across treatment arms at time of randomization into IMPROVE.  

- **What will the pill count be assessing? Fluconazole or 1HP or both or drugs for other co-morbidities? These need to be stated clearly. Assessment of adherence to all medications is key as it has an effect or impact on treatment outcomes and may be confounders. We are looking at integration of management. Another reason why we should monitor for adherence on all medications.**

Pill counts refers to 1HP only. This has been clarified in the text.  

- **What drug will the patient be taking after completing the 10 week continuation phase? This should be stated in the study procedure sub-section as the study follow up period is for 18 weeks.**

200mg of fluconazole. This has been added to the text. **“We anticipate that ~1/3 of participants will be ART naïve, these patients will start ART at week 4–6, ~1/3 of participants will be on ART but have clinical/immunological/virological failure”**

**What are these anticipations based on? What is the evidence for this or is it an educated guess? Is it from literature or from figures at the study sites? Too much has been summarized in this tables and needs to be explained under the methods section above** This is based on our experience working at the study sites.

**Inpatient management:**

**“ Following hospital discharge participants will be followed-up every two weeks until week-18”**

**What measures are the authors putting in place to reduce lost to follow up during the follow-up period? All these should be stated clearly? Are there incentives such as tokens for transport purposes for patients to increase their chances of coming for review? Are information on their phone contacts and those of caregivers being taken to do follow-up? Who will be one doing the follow-up? All these should be clearly outlined in the protocol**   Thank you for these points. The following have been included within the follow-up section:

- At time of discharge contact details for participants are recorded to facilitate follow-up.
- Follow-up visits are performed by the IMPROVE study team.
- Participants are reimbursed for travel to outpatient visits.

 
**Outpatient management (point 4):**

**The study physician is part of the research group and there must be a guideline or protocol to be followed for ruling out active TB. This should be stated. It cannot be at the discretion of the study physician as it affects the rigorousness of the study. **   Thank you for this point. The following has been added:   At each study visit IMPROVE participants will undergo systematic TB screening using WHO TB 4-symptom screen (WHO4SS) and if screen positive will undergo microbiological investigation with urine Alere-LAM, and sputum Xpert Ultra if able to produce sputum.  If microbiological investigations are negative this will be followed up with a chest radiograph to further investigate for TB.

- A diagnosis of confirmed TB disease will be assigned in cases with any positive microbiological TB test at any point during the study period, whether TB treatment is initiated or not.
- Participants treated for tuberculosis but without microbiological confirmation will be assigned by a clinical end-point adjudication committee as either probable – if radiological and/or clinical features (which could include consideration of treatment response) are considered compatible with active TB – or possible – if radiological and clinical features are not deemed sufficient for a diagnosis of active TB (which could also include a lack of information such as a missing chest radiograph or radiograph report).
- Participants not treated for active TB will be assigned as ‘not tuberculosis’ except in the circumstance in which TB treatment is withheld from an individual determined to have active TB but in whom treatment is not deemed appropriate (for example in end-of-life care) when they will be assigned as confirmed, probable or possible according to criteria above.

**Sample size:**

- **What is the justification for this sample size? How was it calculated and what were the basis for the calculation?**
- **Since there are 2 groups, is it 205 per group OR the 2 groups make up the 205?**

1HP treatment completion was 97% in the BRIEF-TB trial in the outpatient setting; as the key trial of 1HP amongst PLHIV these were the primary data to inform our power calculations. Given that our cohort have more advanced HIV disease, and given the possibility of poorer adherence in the inpatient arm, we modelled a range of power calculations, but assuming an 80% TB-disease free completion rate in the outpatient 1HP initiation arm, a total sample size of 205 would give us 80% power to determine whether early 1HP led to non-inferior TB-disease free completion rates. **Power calculations detailing a range of possible sample sizes (overall, assuming 1:1 randomisation) depending on non-inferiority margin and TB-free 1HP completion rates.** **TB-free treatment completion in standard 1HP arm (outpatient setting)** **80%** **82%** **84%** **86%** **90%** **Non-inferiority margin** **10%** 460 425 385 345 260 **12%** 319 295 268 240 180 **15%** **205 ** 189 172 154 117 Sample sizes are based on 80% power, with a one sided 95% confidence interval, and allowing for 5% LTFU and 10% post-randomisation mortality at 18-weeks.

- **Will be good to state the formula used or the software used and the reference for readers to follow. And this should not be mixed with the statistical analysis methods**

  This has been added. Power calculations were conducted using Sealed Envelope Ltd. 2012. Power calculator for binary outcome non-inferiority trial. [Online] Available from: https://www.sealedenvelope.com/power/binary-noninferior/ [Accessed Wed May 01 2024].

  

- **What is the basis for selection of 5% as the loss to follow up rate and not 10% or 15%?**

This is based on our experience conducting clinical studies across the study sites since 2006. Thank you again for the review Very best Dr Jayne Ellis

# Reviewer report: Reviewer response for version 1

**DOI:** 10.21956/wellcomeopenres.21407.r73746

**Published:** 2024-04-26

**Authors:** Ajay Rangaraj

**Affiliations:** 1 Department of HIV, Hepatitis and STIs, World Health Organisation, Geneva, Switzerland

Thank you for the opportunity to review this well written manuscript. 

1. Please do include the rationale for why 18 weeks was chosen as an appropriate end-time period for the study. 
2. It would be a great inclusion if the study can report on post-discharge outcomes - not just for TB, but also for CM. And to  report the outcomes more systematically such as post discharge status (functional status), whether re-linked to care for ART, LTFU etc.,  as it is a highly relevant issue in AHD. 
3. It would be good to mention what regimen is being used in the antifungal treatment - as they are not all equal - particularly if conventional amphotericin-B is being used and/or then single high dose liposomal amphotericin. This would also mean the study should track kidney parameters. 
4. Under "Consent"- please do consider rephrasing the word ïlliterate" - could perhaps be reworded to "those that are unable to read or write". 
5. The study's primary endpoint is "Treatment completion is defined as participant reported adherence to >90% of the study medications, to be completed within 6-weeks from treatment initiation." - I think I understand this as all the study medications - could you please clarify this? 
  Also if the individual is not conscious during the inpatient stay - how would adherence be calculated? Or would it be the case that it would be 100%?
6. Is there any intention to look at resistance development in the study - would be important to mention if or if not. 
7. Could there have been additional arms in this trial to evaluate 3HP? Was there a rationale to not include it? 

Is the study design appropriate for the research question?

Yes

Is the rationale for, and objectives of, the study clearly described?

Yes

Are sufficient details of the methods provided to allow replication by others?

Yes

Are the datasets clearly presented in a useable and accessible format?

Not applicable

Reviewer Expertise:

I work on synthesising evidence for and coordinating global treatment guidelines updates for HIV treatment and care - particularly ART, Advanced HIV disease, cryptococcal meningitis, histoplasmosis and other opportunistic infections.

I confirm that I have read this submission and believe that I have an appropriate level of expertise to confirm that it is of an acceptable scientific standard, however I have significant reservations, as outlined above.

# Response

**Published:** 2024-05-09

**Authors:** Jayne Ellis

**Affiliations:** Infectious Diseases Institute, Uganda

**Dear Ajay Rangaraj** **Thank you for taking the time to review the IMPROVE protocol - your feedback is very much appreciated. Please find below responses to each of your comments raised: **

- **Please do include the rationale for why 18 weeks was chosen as an appropriate end-time period for the study. **

This end-time was chosen because the risk of mortality, opportunistic infections and immune reconstitution inflammatory syndrome (IRIS) events (including TB-IRIS) are considerably lower beyond 18-weeks following immune reconstitution with predominately DTG-based ART initiation/switch at 4-6 weeks post cryptococcal meningitis diagnosis.  

- **It would be a great inclusion if the study can report on post-discharge outcomes - not just for TB, but also for CM. And to  report the outcomes more systematically such as post discharge status (functional status), whether re-linked to care for ART, LTFU etc.,  as it is a highly relevant issue in AHD. **

In parallel to the IMPROVE trial, where consent is given, study participants are also followed up longitudinally as part of our open cohort study (Improving Diagnostics and Neurocognitive Outcomes in HIV/AIDS-related Meningitis) which collects post-discharge outcomes including neuro-cognitive assessments at annual follow-up visits.  

- **It would be good to mention what regimen is being used in the antifungal treatment - as they are not all equal - particularly if conventional amphotericin-B is being used and/or then single high dose liposomal amphotericin. This would also mean the study should track kidney parameters. **

All IMPROVE participants receive amphotericin-based induction anti-fungal therapy. The induction regimen received however, does depend upon drug availability (liposomal amphotericin vs. amphotericin deoxycholate) and whether the participant received induction anti-fungal therapy as part of a randomized controlled trial. Given this is a randomized trial however, we do anticipate any differences in induction regimens to be well balanced between IMPROVE trial arms. Study participants have both in-hospital, and post-discharge protocolized safety monitoring blood tests including kidney parameters, liver function, and complete blood counts.  

- **Under "Consent"- please do consider rephrasing the word illiterate" - could perhaps be reworded to "those that are unable to read or write". **

Thank you, this has been amended in the updated version of the protoc.  

- **The study's primary endpoint is "Treatment completion is defined as participant reported adherence to >90% of the study medications, to be completed within 6-weeks from treatment initiation." - I think I understand this as all the study medications - could you please clarify this? **

Yes this means that a participant must complete >90% of both isoniazid and rifapentine within 6-weeks from initiation of the 1HP regimen i.e. they cannot miss more than 3 days of treatment. **Also if the individual is not conscious during the inpatient stay - how would adherence be calculated? Or would it be the case that it would be 100%?** Participants are randomised into the IMPROVE RCT at time of discharge (other than in instances, where the participant remains an inpatient for ≥14 days, randomisation will occur on day 14 rather than on the planned day of discharge), therefore participants rarely have reduced level of consciousness during the IMPROVE trial. In cases where a participant does have a reduced level of consciousness such that they are unable to self-medicate, a naso-gastric tube is inserted to ensure adherence with both IMPROVE study drugs, and with anti-fungal medications, and nutritional support.

  

- **Is there any intention to look at resistance development in the study - would be important to mention if or if not. **

In cases of culture-proven incident TB disease (e.g. blood culture positive TB-cases) drug susceptibility testing is performed. This has been added to the manuscript.

- **Could there have been additional arms in this trial to evaluate 3HP? Was there a rationale to not include it? **

Within IMPROVE, we are specifically interested in investigating whether TB preventive therapy (TPT) can be initiated as an inpatient for patients with advanced HIV disease and cryptococcal meningitis, such that TPT completion (and sterilisation of latent TB infection) can be achieved prior to ART initiation/switch at ~6-weeks post cryptococcal meningitis diagnosis. We hypothesize that TPT completion prior to ART initiation/switch will reduce the risk of unmasking TB-IRIS events in this population. Whilst 3HP would have been an interesting comparator arm (for example reduced pill-burden, +/- reduced risk of drug-drug interactions as compared to 1HP in this group), it would not allow for TPT completion prior to ART initiation/switch. Furthermore the limited budget and therein sample size, necessitated a two-arm trial only.   Many thanks again for the review Very best Dr Jayne Ellis
